# Ketogenic diet therapy leads to antiseizure medication reduction in children and adults with drug‐resistant epilepsy

**DOI:** 10.1111/cns.14854

**Published:** 2024-07-17

**Authors:** Fang He, Lingqi Ye, Leilei Wang, Jiping Zhou, Xiaotong Shao, Pu Miao, Shan Wang, Hong Li, Yao Ding, Shuang Wang

**Affiliations:** ^1^ Department of Nutrition, Second Affiliated Hospital, School of Medicine Zhejiang University Hangzhou China; ^2^ Department of Neurology, Epilepsy Center, Second Affiliated Hospital, School of Medicine Zhejiang University Hangzhou China; ^3^ Department of Neurology Wayne State University/Detroit Medical Center Detroit Michigan USA; ^4^ Department of Radiology, Second Affiliated Hospital, School of Medicine Zhejiang University Hangzhou China; ^5^ Department of Pediatrics, Second Affiliated Hospital, School of Medicine Zhejiang University Hangzhou China

**Keywords:** adults, anti‐seizure medications, drug‐resistant epilepsy, ketogenic diet, withdrawal

## Abstract

**Aims:**

This study aimed to evaluate the safety of reducing or withdrawing anti‐seizure medications (ASMs) in a cohort comprising both adults and children with drug‐resistant epilepsy (DRE) undergoing ketogenic diet therapy (KDT).

**Methods:**

We conducted a comprehensive analysis of clinical profiles in adults and children with DRE who had adhered to KDT for at least 6 months. Successful withdrawal or reduction of an ASM was defined as discontinuation or dose reduction without subsequent resumption or increase and without initiation of any new ASM throughout the entire follow‐up period. Changes in the ASM load were calculated specifically for adult patients.

**Results:**

The study enrolled 56 participants (34 children and 22 adults) with DRE, with 64.3% achieving successful withdrawal of at least one ASM. The probability of ASM withdrawal remained consistent for children (64.7%) versus adults (63.6%), as well as for responders (62.5%) versus non‐responders (68.8%), and it was not associated with other clinical factors. Early ASM reduction (including withdrawal) after diet initiation occurred in 15 patients (26.8%), with treatment outcomes comparable to those of the remaining participants. Among the 22 adults, the mean values of ASM load reduced by 24.5%, with a similar magnitude observed for responders (24.2%) versus non‐responders (25.1%). In addition, adults tend to have a slower elevation in serum ketone levels compared to children.

**Conclusion:**

This study demonstrates the safe achievability of ASM withdrawal through KDT in most patients with DRE, irrespective of age or seizure frequency reduction.

## Graphical abstract

This study provides further evidence supporting safety of anti‐seizure medications reduction or withdrawal during ketogenic diet therapy in both adult and children patients with drug‐resistant epilepsy. Additionally, it highlights that early anti‐seizure medication reduction does not impact long‐term outcomes of ketogenic diet therapy.

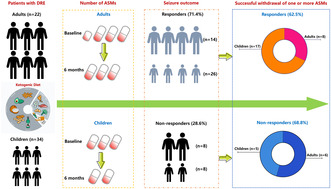

## 1 INTRODUCTION

Epilepsy manifests as a chronic neurological disorder characterized by recurrent seizures, stemming from episodes of excessive and synchronous neuronal activity in the brain.^1^, ^2^ The cornerstone of treatment for this condition lies in anti‐seizure medications (ASMs). However, approximately 30% of patients with epilepsy face challenges in achieving complete seizure control through ASMs.^3^, ^4^ Individuals with drug‐resistant epilepsy (DRE) often endure high doses of ASMs, leading to adverse reactions such as impacting vigilance, psychiatric symptoms, cognitive dysfunction, social behavior, and teratogenicity, especially for females of reproductive age.^5^, ^6^, ^7^, ^8^, ^9^ The burden of these adverse reactions and uncontrolled seizures significantly compromises the quality of life and social functioning of patients.

While several non‐pharmacological treatments, such as neuromodulation and surgical resection, have demonstrated efficacy in controlling seizures in patients with DRE, their applicability to all patients is limited. Ketogenic diet therapy (KDT), centered on a high‐fat and carbohydrate‐restricted diet,^10^, ^11^ has proven effective in controlling epilepsy and offers adaptability across various dietary cultures. Our primary study was the pioneering effort to establish the safety and effectiveness of KDT for DRE in the Chinese adult and pediatric population, revealing a high retention rate of 79% at 6 months after diet initiation.^12^ The benefits of KDT extend beyond seizure control, encompassing improvements in cognitive function, vigilance, and attention.^13^ Despite the lifestyle constraints it imposes, KDT is highly recommended due to its non‐invasiveness, favorable treatment outcomes, and good tolerance.

Another notable advantage of KDT is the reduction of ASMs. In a prospective study, 74% of children on the diet achieved a reduction in ASMs over a 12‐month period.^14^ Kossoff et al.^15^ reported a 57% reduction in ASMs at the first month and a 65% reduction at the 6‐month mark in a pediatric population, accompanied by concurrent improvements in vigilance and cognition, regardless of the timing of ASM reduction during treatment. A recent United Kingdom‐based study revealed that 63% of children with epilepsy could discontinue at least one ASM while undergoing KDT.^16^ While various studies have explored ASM adjustments in children during KDT, limited data exist regarding medication adjustments in adults. Notably, differences in drug metabolism between adults and children, along with the feasibility of quantifying ASM load in adults but not in children, underscore the importance of analyzing the reduction and withdrawal of ASMs in adult patients undergoing KDT.

This study comprehensively evaluates the success rate and safety of ASM withdrawal on KDT in a mixed cohort of adult and pediatric patients with DRE. We compare the medication reduction rate in adults with that in children and systematically analyze factors potentially influencing successful drug withdrawal on KDT, such as age, epilepsy type, and treatment response. Furthermore, we quantitatively measured the dynamic change of ASM load in adult patients undergoing KDT.

## 2 METHODS

### 2.1 Study population

Patients diagnosed with DRE who underwent KDT from November 2017 to October 2022 were included in the study. Drug‐resistant epilepsy was defined as the persistence of seizures after “adequate trials of two tolerated and appropriately chosen and used anti‐seizure medication schedules.”^17^ Patients were categorized into pediatric (<18 years old) and adult (≥18 years old) groups. The inclusion criteria were as follows: (1) diagnosed with DRE; (2) experiencing at least one uncontrolled debilitating seizure per month or status epilepticus; (3) ineligibility for significant benefit from resective surgery, refusal to undergo surgery, or reluctance to receive neuromodulation; (4) treated with KDT for at least 6 months and followed by ketogenic diet teams in our center. The classic ketogenic diet, with a 4:1 ratio of fats to proteins and carbohydrates, was administered during hospitalization under the guidance of the ketogenic diet team. Energy requirements and macronutrient targets were calculated by a dietitian based on the baseline anthropometric data. Personalized ketogenic diet recipes were developed and updated monthly according to patient feedback. All pediatric and adult patients were initiated on a 4:1 ratio of KD, with the exception of two children who began with a 2:1 ratio.

Clinical history, video‐electroencephalogram (EEG) findings, and neuroimaging data were collectively reviewed in a multidisciplinary conference. Dietitian‐conducted remote interviews, facilitated through a domestic telemedicine system, telephone, or WeChat (a Chinese social media platform), occurred at least once a week. Within the first 6 months following KD initiation, we analyzed adjustments made to ASMs and evaluated treatment outcomes.

### 2.2 Definitions and adjustment of ASMs

The decision to prioritize the reduction of a particular medication is based on factors encompassing the patient's seizure control, drug side effects, the impact of the medication on KD, and the patient's individual needs. In this study, the primary outcome was the proportion of patients in whom at least one ASM was successfully withdrawn. Successful withdrawal or reduction of an ASM was defined as a period exceeding 3 months, during which the medication was permanently discontinued or reduced in dosage without the necessity to restart, increase, or initiate any new ASMs throughout the entire follow‐up period (≥9 months).^16^ The treatment outcome of KDT was classified as non‐response when the frequency of seizures decreased by less than 50% compared to baseline and as response if the reduction rate was equal to or greater than 50%. The seizure severity after KDT was evaluated according to the patient's seizure diary and was compared to it before treatment. An alleviation in seizure severity was observed if at least one of the following criteria was met: (1) ≥50% reduction of seizure duration; (2) less impaired consciousness during seizures; (3) prominent improvement in the post‐ictal state; (4) disappearance of clustered seizures. Changes in seizure frequency and severity were determined based on the patient's medical records before initiating KD, as described previously.^12^ The terms “early ASM reduction” and “other” specified the commencement of ASM reduction (including ASM withdrawal) during the periods 0–1 month and 1–6 months after initiating the diet, respectively.^15^ Adjustment of ASMs was personalized for each patient, considering clinical factors such as seizure frequency, potential side effects, and requests from patients or their families. Tapering periods typically extended beyond 4–6 weeks. In cases where seizures worsened over a period longer than 72 h during the tapering process, it was recommended to return the ASM to its previous dose for the patient.

### 2.3 Calculation of ASM load

The ASM load in adults was computed following the 2020 World Health Organization Center for Drug Statistics Methodology ATC/DDD Index, using the formula “individual ASM dosage per day divided by defined daily dose (DDD).”^18^ In patients taking multiple ASMs, the sum of all ratios was calculated and used for analysis. For instance, a patient receiving 1500 mg of valproate (VPA) per day (with a defined DDD of VPA valproate set at 1500 mg) would have an ASM load of 1.0. If the patient were taking 1500 mg of VPA and 150 mg of lamotrigine (LTG) per day (with a DDD of LTG set at 300 mg), the combined ASM load would be 1.5.

### 2.4 Statistical analysis

The quantitative variables demonstrated a non‐normal distribution and were presented as median and interquartile range (IQR). Categorical variables were expressed as numbers and percentages. We utilized Wilcoxon's paired test to analyze the number of ASMs before and after KD treatment. Additionally, repeated measures analysis of variance (ANOVA) was conducted to compare the ASM load at different time points in adult patients and used to test serum ketone levels at different time points. An unpaired *t*‐test was used to compare serum ketone level between adults and children. For multiple comparisons, Bonferroni correction was applied. Categorical data underwent analysis using Fisher's or chi‐square test. Factors predicting successful ASM withdrawal were assessed through logistic regression. GraphPad Prism version 8 (San Diego, CA) was employed to generate plots illustrating the loads of ASMs over different periods. The significance level for all tests was set at *p* < 0.05. Statistical analyses were performed using SPSS version 17.0 (Chicago, IL).

## 3 RESULTS

### 3.1 Cohort characteristics

Between January 2017 and October 2022, a total of 67 patients (28 adults and 39 children) with DRE initiated classic KDT. Among these, 56 patients (60.7% female) meeting the inclusion criteria were enrolled in the study. Eleven patients discontinued KDT within 6 months or were lost to follow‐up for the following reasons: unfavorable response to KDT (3/11, 27.3%), refusal for follow‐up (3/11, 27.3%), unacceptance of diet restriction (2/11, 18.2%), lack of adequate family/medical support (2/11, 18.2%), and medical complications unrelated to the diet treatment. The baseline demographic data and clinical details of our cohort are displayed in Table 1. The cohort comprised 22 adults (24.5, IQR 19.8–29.3 years old) and 34 children (10.4, IQR 7.2–14.0 years old). The response rate was 62.5% at 3 months and 71.4% at 6 months after the initiation of the diet, respectively. Six children achieved seizure freedom, while none of the adults reached this outcome. About 66.1% of the 56 patients experienced alleviation of seizure severity after undergoing the KDT. The results are detailed in Table S1. No differences in response rates were observed among the three categories of etiology: genetic (69.2%), structural (73.3%), and unknown (69.2%).

**TABLE 1:** Demographic data of included patients on ketogenic diet therapy.

	Adults, *n* = 22	Children, *n* = 34	Total, *n* = 56
Gender, male, *n* (%)	6 (27.3%)	16 (47.1%)	22 (39.3%)
Epilepsy duration, year, median (IQR)	7.0 (2.0, 14.9)	4.0 (2.4, 6.5)	4.3 (2.1, 9.0)
Age at diet start, year, median (IQR)	24.5 (19.8, 29.3)	10.4 (7.2, 14.0)	14.0 (10.0, 21.0)
Epilepsy type, *n* (%)			
Generalized	5 (22.7%)	11 (32.4%)	16 (28.6%)
Focal	17 (77.3%)	23 (67.6%)	40 (71.4%)
Etiology, *n* (%)			
Structural	13 (59.1%)	17 (50.0%)	30 (53.5%)
Genetic	3 (13.6%)	10 (29.4%)	13 (23.2%)
Unknown	6 (27.3%)	7 (20.6%)	13 (23.2%)
Baseline seizure frequency, *n* (%)			
Daily	8 (36.4%)	8 (23.5%)	16 (28.6%)
Weekly	7 (31.8%)	11 (32.4%)	18 (32.1%)
Monthly	7 (31.8%)	15 (44.1%)	22 (39.3%)
Response rate at 3 months, *n* (%)	11 (50.0%)	24 (68.6%)	35 (62.5%)
Response rate at 6 months, *n* (%)	14 (63.6%)	26 (76.5%)	40 (71.4%)
Number of ASMs, median (IQR)	3.5 (3.0, 4.0)	3.0 (3.0, 4.0)	3.0 (3.0, 4.0)

### 3.2 ASM withdrawal

Successful ASM withdrawal was accomplished in 36 patients (64.3%), including 14 adults (8 responders vs. 6 non‐responders) and 22 children (17 responders vs. 5 non‐responders), constituting 62.5% of responders and 68.8% of non‐responders. No statistically significant difference was noted between adults and children (63.6% vs. 64.7%, *p* = 0.935). Withdrawal of one ASM was observed in 25 patients (11 adults and 14 children), and two ASMs in 11 patients (3 adults and 8 children). No patient achieved the withdrawal of all ASMs during KDT. The most frequently withdrawn ASMs were VPA (22/32, 68.6%), topiramate (TPM) (6/16, 37.5%), and perampanel (2/8, 25%) (Table 2). At 6 months after the initiation of KDT, the median number of ASMs in use was 2.0 (IQR 2.0–3.0), significantly lower than before KDT (3.0, IQR 3.0–4.0; *p* < 0.0001). This statistical significance was also observed in both adults [3.0 (IQR 2.0–3.25) vs. 3.5 (IQR 3.0–4.0), *p* < 0.0001] and children [2.0 (IQR 2.0–3.0) vs. 3.0 (IQR 3.0–4.0), *p* < 0.0001]. Results of the univariable analysis revealed that epilepsy type was significantly associated with the chance of successful ASM withdrawal (*p* = 0.047) (Table 3). However, the multivariable logistic regression model showed no significant associations between successful ASM withdrawal and clinical factors (Table S2).

**TABLE 2:** Anti‐seizure medication withdrawal at different time point and in different patients during ketogenic diet therapy.

Number of cases on ASM (*n*)	Number of cases with ASM withdrawal			Number of cases with ASM withdrawal		
	1 month	2–3 months	4–6 months	Children	Adults	Total
Valproate (*n* = 32)	12 (37.5%)	6 (18.8%)	4 (12.5%)	16 (64.0%)	6 (85.7%)	22 (68.6%)
Topiramate (*n* = 16)	1 (6.3%)	0 (0.0%)	5 (31.3%)	4 (36.4%)	2 (40.0%)	6 (37.5%)
Perampanel (*n* = 8)	0 (0.0%)	2 (25.0%)	0 (0.0%)	1 (25.0%)	1 (25.0%)	2 (25.0%)
Lamotrigine (*n* = 27)	3 (11.1%)	3 (11.1%)	0 (0.0%)	5 (33.3%)	1 (8.3%)	6 (22.2%)
Lacosamide (*n* = 14)	0 (0.0%)	2 (14.3%)	0 (0.0%)	1 (12.5%)	1 (16.7%)	2 (14.3%)
Levetiracetam (*n* = 40)	1 (2.5%)	0 (0.0%)	3 (7.5%)	2 (8.3%)	2 (12.5%)	4 (10.0%)
Clonazepam (*n* = 21)	1 (4.8%)	1 (4.8%)	0 (0.0%)	1 (10.0%)	1 (9.1%)	2 (9.6%)
Carbamazepine (*n* = 11)	0 (0.0%)	0 (0.0%)	1 (9.1%)	0 (0.0%)	1 (20.0%)	1 (9.1%)
Oxcarbazepine (*n* = 23)	0 (0.0%)	1 (4.3%)	1 (4.3%)	1 (7.8%)	1 (10.0%)	2 (8.6%)
(*n* = 2)	0 (0.0%)	0 (0.0%)	0 (0.0%)	0 (0.0%)	0 (0.0%)	0 (0.0%)

**TABLE 3:** Predictors for successful ASM withdrawal on univariate univariable analysis (*n* = 56).

Clinical factors	ASM withdrawal (*n* = 36)	No ASM withdrawal (*n* = 20)	*p* Value
Age at the start of KDT, year, median (IQR)	14.5 (10.0, 20.0)	13.2 (10.0, 23.3)	0.791
Epilepsy duration, year, median (IQR)	4.0 (2.1, 7.2)	4.8 (2.1, 10.8)	0.585
Etiology, *n* (%)			
Known	28 (77.8%)	15 (75.0%)	0.814
Unknown	8 (22.2%)	5 (25.0%)	
Epilepsy type, *n* (%)			
Generalized	7 (19.4%)	9 (45.0%)	0.047*
Focal	29 (80.6%)	11 (55.0%)	
Response to KDT at 3 months, *n* (%)			
Responders	27 (75.0%)	13 (65.0%)	0.429
Non‐responders	9 (25.0%)	7 (35.0%)	
Number of ASMs at the start of KDT, median (IQR)	3.0 (3.0, 4.0)	3.0 (3.0, 4.0)	0.202

Abbreviations: ASM, anti‐seizure medication; Asterisk: The etiology of epilepsy was categorized into two types of “known” and “unknown” for easier analysis; KDT, ketogenic diet therapy.

* *p* < 0.05.

### 3.3 Outcomes of early ASM reduction group

During the initial month of KDT initiation, 26.8% (15/56) of the cohort experienced early withdrawal of ASM. Nevertheless, early ASM reduction was attained in 34 out of 56 cases (60.7%), comprising 11 adults and 23 children (Table 4). When comparing the early ASM reduction group to the others, no significant difference was observed in treatment response at either 3 months (*p* = 0.323) or 6 months (*p* = 0.100) after diet initiation. Additionally, no differences were found in either adults (3 months, *p* = 0.670; 6 months, *p* = 0.699) or children (*p* = 0.692; *p* = 0.388). Treatment response remained very similar at 6 months compared to 3 months after diet initiation, both in the early ASM reduction group (*p* = 0.272) and in others (*p* = 0.761). Similar results were also found in the alleviation of seizure severity, regardless of comparing the early ASM reduction group with others at 3 months (*p* = 0.379) or 6 months (*p* = 0.143), and comparing 3 months with 6 months in the early ASM reduction group (*p* = 0.078) or others (*p* = 0.365). Our data demonstrated that early ASM reduction did not impact outcomes, and the response to KDT remained stable during the 3–6 month period.

**TABLE 4:** ASM reduction and seizure outcome.

Outcome of KDT	Early ASM reduction			Others			*p* Value
	Children (*n* = 23)	Adults (*n* = 11)	Total (*n* = 34)	Children (*n* = 11)	Adults (*n* = 11)	Total (*n* = 22)	
>50% seizure reduction at 3 months	17	6	23	7	5	12	0.323
>50% seizure reduction at 6 months	19	8	27	7	6	13	0.100
Alleviated seizure severity at 3 months	14	4	18	5	4	9	0.379
Alleviated seizure severity at 6 months	19	6	25	7	5	12	0.143

*Note*: In this part, the number of reduced ASMs included withdrawal and reduction in ASM dosage. *p* Value represented the comparison between total Early ASM reduction group and total “others” group.

Abbreviations: ASM, anti‐seizure medication; KDT, ketogenic diet therapy.

In our cohort, approximately 30% (17/56) of patients experienced temporary seizure worsening after ASM reduction. After undergoing 3 days of close observation, the seizure frequency in most of the patients fell without further medication adjustment, except for one young boy with Dravet syndrome who exhibited a significant increase in seizure frequency after reduction of VPA. This prompted us to add VPA back into the previous dosage.

**FIGURE 1:**
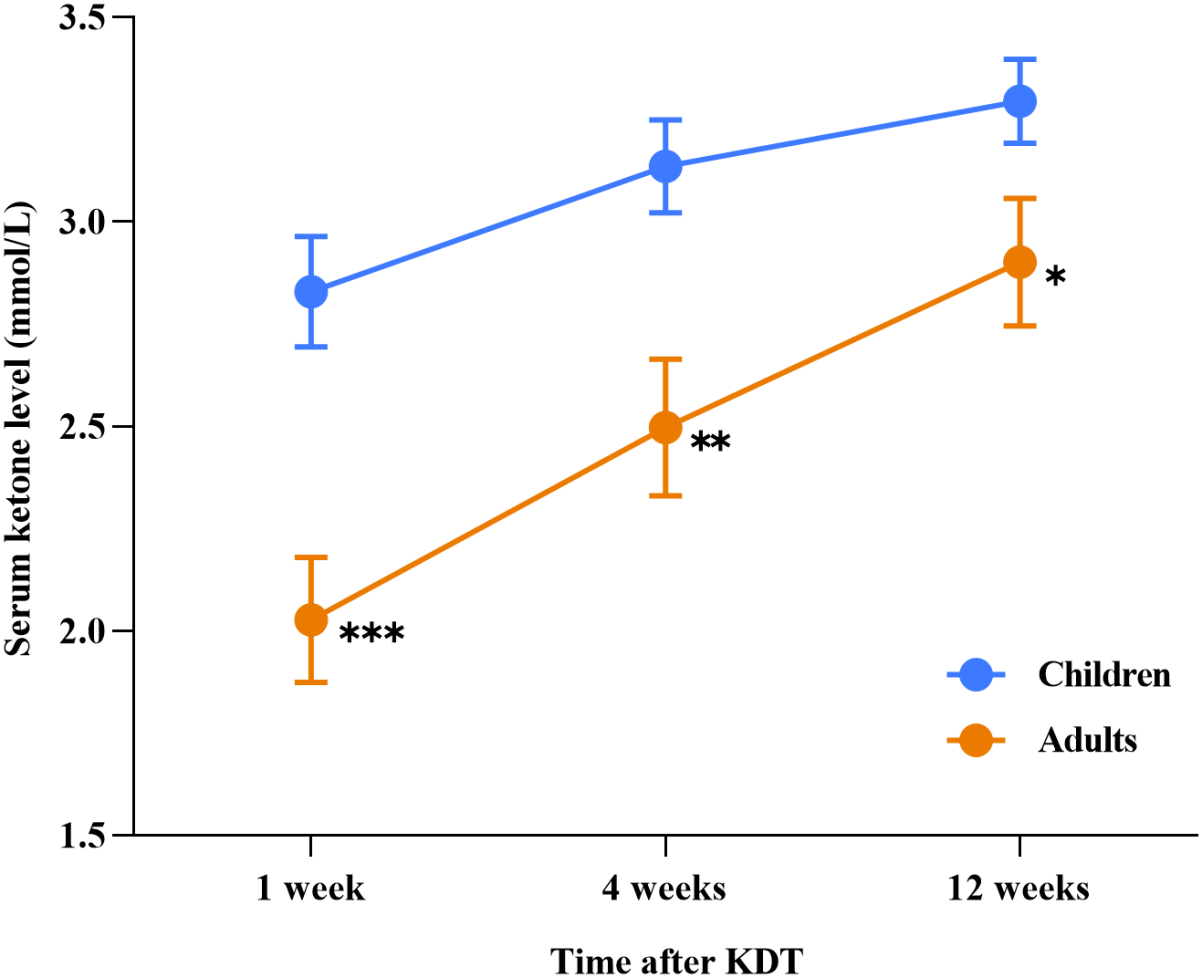
Difference in serum ketone level between adults and children at various time points. It was observed that adults tend to have a slower elevation in serum ketone levels compared to children. This also shows that serum ketone levels varied at various time points (*p* < 0.0001) and that there was significant interaction between the times and groups (*p* = 0.013). The serum ketone level is presented as mean ± SEM. *p < 0.05; **p < 0.01; ***p < 0.001. KDT, ketogenic diet therapy.

We also calculated the mean level of serum ketone in children and adults at different time points within the 12 weeks after starting KDT, except for three individuals (one child and two adults) who did not have timely measurements of serum ketone (Figure 1). The mean serum ketone values were 2.03 ± 0.68 and 2.83 ± 0.77 mmol/L in the adults and children at 1 week after KDT (*p* < 0.0001), 2.49 ± 0.75 and 3.14 ± 0.65 mmol/L at 4 weeks after KDT (*p* = 0.002), and 2.90 ± 0.69 mmol/L and 3.29 ± 0.59 mmol/L at 12 weeks after KDT (*p* = 0.034), respectively. In addition, a sustaining rise of the serum ketone levels had been observed over the 12‐week period of KDT, no matter in the adult or the children group.

### 3.4 ASM load in adults

**FIGURE 2:**
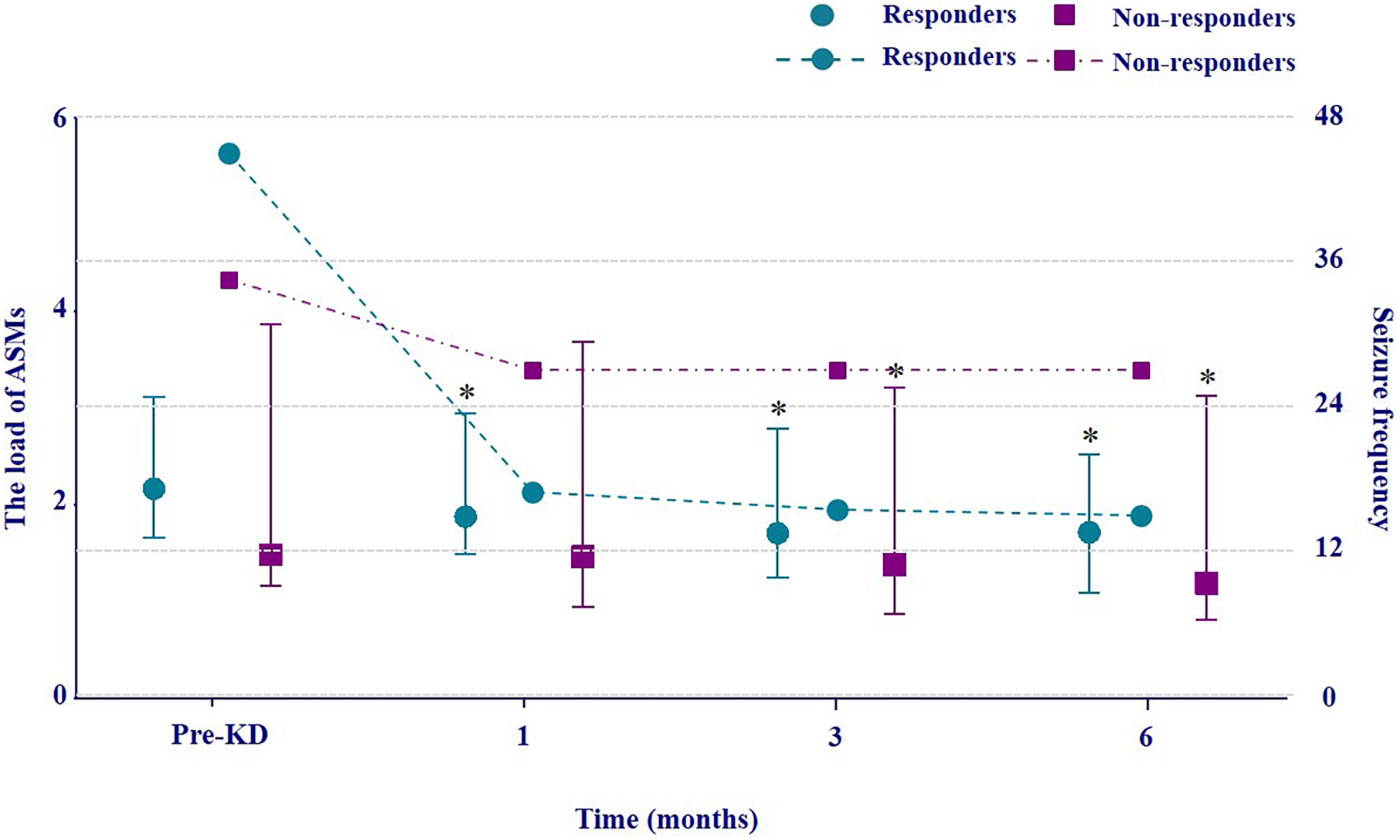
Change of anti‐seizure medication (ASMs) loads and seizure frequency in adults during ketogenic diet therapy (KDT). The horizontal *x*‐axis represents different time points before and after ketogenic diet treatment. Median values of ASM loads with interquartile ranges (left *y*‐axis) are represented by green circles and purple squares. The green circles and purple squares connected by dotted lines represent seizure frequency (right *y*‐axis), which is averaged for each month. Green color represents responders and purple color represents non‐responders.*p < 0.05.

The ASM load was calculated in all adult patients (14 responders and 8 non‐responders). At 6 months, the values of ASM load were reduced in 13 responders and 8 non‐responders. In the subgroup analysis, the values of ASM load in responders (*p* = 0.009, *p* = 0.005) and non‐responders (*p* = 0.042, *p* = 0.013) were both significantly lower compared to the pre‐treatment level at 3 and 6 months after diet initiation; whereas only in responders, the ASM load was significantly reduced at 1 month after diet initiation (*p* = 0.042) (Figure 2).Overall, the mean values of ASM load of adult patients decreased by 24.5% at 6 months, including a reduction of 24.2% in the responder group and 25.1% in the non‐responder group. Meanwhile, the overall seizure frequency of the adult group did not increase over the 6‐month period after diet initiation and remained relatively stable for both groups at 1, 3, and 6 months.

## 4 DISCUSSION

In our study, we systematically analyzed the successful withdrawal and reduction of ASM in patients adhering to KDT over 6 months. Our study provides further evidence that ASMs can be safely withdrawn during KDT in patients with DRE. Additionally, our study highlights that early ASM reduction does not impact long‐term outcomes of KDT.^15^ More than half of the patients successfully achieved withdrawal of at least one medication. The withdrawal rate was comparable between adults (63.7%) and children (64.7%), and this proportion closely aligns with the findings from a previous study in children (62.9%).^16^ In the adult patients in our cohort, the ASM load was equally reduced in both responders and non‐responders.

Patients with DRE commonly employ combinations of two or more ASMs, heightening the risk of experiencing side effects and imposing an economic burden. In our cohort, a majority of patients were prescribed three or more ASMs before initiating KDT. Roughly half of the adult patients were taking four or more ASMs. Adjusting ASMs in patients with DRE is a complex dynamic process fraught with challenges. Inappropriate reduction or withdrawal of ASMs could lead to seizure worsening, manifesting as either a temporary or long‐term effect.

The KDT has demonstrated effectiveness in controlling seizures across different types of epilepsy and etiologies in both children and adults.^12^, ^19^ No difference was found between the response rate of KDT at 3 or 6 months, indicating stable efficacy over time. The KDT provides particular benefits in special circumstances, such as in patients with glucose transporter type 1 deficiency syndrome and those experiencing epileptic spasms‐tonic seizures, leading to a higher success rate in ASM withdrawal.^19^, ^20^ Although such patients were not included in our cohort, our study still verified the robust efficacy of KDT in both adults and children. We observed that adults tend to have a slower elevation in serum ketone levels compared to children, which is unsurprising, but very interesting and practical for clinicians to make individual protocols for KDT. Since children have less glycogen stored than adults, they usually rely more on fat oxidation than adults, with increased mobilization of free fatty acids, glycerol release, and growth hormone levels in preadolescent children providing evidence for this.^21^ This may explain the faster ketosis in children. Our data revealed that the chance of successful ASM withdrawal was equal for responders and non‐responders and was not necessarily associated with clinical factors. The underlying mechanism that explains the broad‐spectrum efficacy of KDT is rather multifaceted. Consistent with a previous study,^16^ we also observed seizure worsening when reducing ASMs, but it was usually temporary and rarely led to the consequence of adding back the medications. This suggests that the temporary seizure worsening observed during medication reduction does not impact the long‐term outcome of KDT.

Ketones and ASMs interact with each other in various aspects. KD induces a wide range of metabolic changes in the body, which may interact with the absorption, distribution, metabolism, and elimination of ASMs.^22^ Some studies suggested that the serum concentrations of several ASMs are significantly reduced during KDT.^22^, ^23^ Conversely, other studies indicated that no significant changes were observed in the serum concentrations of these ASMs during KDT.^24^, ^25^ Overall, previous findings indicate that KDT may not influence the pharmacokinetics of ASMs. Meanwhile, ASMs may have impacts on ketone levels or the efficacy of KD.^26^, ^27^, ^28^ Additionally, it has been reported that the effects of KDT may be diminished by LTG or phenobarbital.^27^, ^29^ Thirdly, KD may modulate the pharmacodynamics of ASMs. In a rodent model, it was discovered that acetone could enhance the effectiveness of several ASMs without altering the brain concentration.^30^ Further research is needed to understand how KD impacts the pharmacokinetics and pharmacodynamics of ASMs, particularly in adults.

In our investigation, the most frequently withdrawn ASMs were VPA and TPM. VPA constituted two‐thirds of the total withdrawn ASMs, with a notable trend of discontinuation within the initial month after diet initiation. Despite previous reports endorsing the safety of VPA in KDT, it was accorded priority for reduction or withdrawal due to several considerations. As a short‐chain fatty acid, VPA elevates fatty acid oxidation, potentially inducing hepatotoxicity.^31^, ^32^ Moreover, VPA has been associated with adverse effects on menstruation and pregnancy,^9^ a concern particularly pertinent to our predominantly female cohort, many of whom were in or approaching fertile age.

These factors likely influenced the decision to prioritize the reduction or withdrawal of VPA in our study. Additionally, VPA inhibits the respiratory chain in mitochondria and ketone production,^32^ and we observed a slower ketone production in adults compared to children at the diet initiation. Notably, in some patients, a decrease in VPA resulted in an increase in serum ketone levels, aligning with findings from a prior study.^26^ The second most frequently discontinued drug was TPM, known for its inhibition of carbonic anhydrase and subsequent reduction in serum bicarbonate levels. This property poses a potential risk of metabolic acidosis. Considering that KDT itself can induce acidosis, kidney stones, and weight loss, TPM was prioritized for reduction to mitigate potential side effects and enhance treatment adherence.

The process of ASM reduction and withdrawal demands a comprehensive consideration of various factors, encompassing the efficacy and side effects of ASMs, interactions between ASMs and KD, and effective communication with patients. It is crucial to time and adjust ASM reduction judiciously. We recommend avoiding periods of fluctuating serum ketone levels and considering personalized seizure triggers, such as emotional instability, life events, and the menstrual period in cases of catamenial epilepsy. This approach ensures that early ASM reduction is both feasible and safe. Additionally, as an added aspect of treatment benefits, ASM reduction may further foster treatment adherence.

## 5 LIMITATION

Firstly, this study constitutes a single‐center observational investigation involving a limited cohort. Future endeavors should prioritize randomized controlled studies. Secondly, our assessment did not include the measurement of cognitive function and vigilance, despite some reports of cognitive enhancement from the families. Thirdly, our study lacks the monitoring of serum concentrations of ASMs.

## 6 CONCLUSION

Our data suggests that the withdrawal of ASMs can be safely accomplished during KDT in both adults and children with DRE, irrespective of the reduction in seizure frequency. The mean ASM load experienced a reduction of a quarter in adults. Additionally, our study reinforces the safety of early ASM reduction, highlighting that it may not impact the long‐term outcomes of KDT.

## AUTHOR CONTRIBUTIONS

FH: conceptualization; data curation; formal analysis, investigation; methodology; writing – original draft; writing – review & editing. LY: investigation; methodology; writing – original draft; writing – review & editing. LW: formal analysis; investigation; methodology. JZ: writing – review & editing. XS: data curation; investigation; methodology. PM: data curation; investigation; resources. SW: data curation; investigation; resources. HL: data curation; investigation; methodology. YD: data curation; investigation; resources. SW: conceptualization; investigation; project administration; supervision; validation; writing – original draft; writing – review & editing.

## FUNDING INFORMATION

This study received support from the National Natural Science Foundation of China (grant number: 82171437) and the Major Project Supported by the Natural Science Foundation of Zhejiang Province (grant number: LD24H090003).

## CONFLICT OF INTEREST STATEMENT

The authors declare no conflicts of interest.

## CONSENT TO PARTICIPATE

Each patient or their guardian provided written informed consent.

## CONSENT FOR PUBLICATION

Written informed consent for the publication of medical data was obtained from each patient or their guardian.

## Supporting information


Tables S1–S2.

## Data Availability

The corresponding author had access to all data in the research. Anonymized data would be available upon reasonable request to the corresponding author.
