# Supplementary material for: Ketogenic diet therapy leads to antiseizure medication reduction in children and adults with drug‐resistant epilepsy
Source: CNS Neurosci Ther. 2024 Jul 17;30(7):e14854. doi: 10.1111/cns.14854 (PMC11255014; doi:10.1111/cns.14854)
Supplement: Supplementary file 1 — Tables S1–S2. [file CNS-30-e14854-s001.docx]

| Table S1. Alleviation in seizure severity in patients with drug-resistant epilepsy | | | | |
| --- | --- | --- | --- | --- |
|  | 1 | 2 | 3 | 4 |
| patient-1 | √ | √ |  |  |
| patient-2 | √ |  |  |  |
| patient-3 | √ | √ | √ |  |
| patient-4 |  |  | √ |  |
| patient-5 |  |  | √ |  |
| patient-6 | √ |  | √ |  |
| patient-7 | √ |  | √ |  |
| patient-8 | √ |  |  |  |
| patient-9 | √ |  |  |  |
| patient-10 |  | √ | √ | √ |
| patient-11 | √ |  |  |  |
| patient-12 |  |  | √ |  |
| patient-13 | √ |  | √ |  |
| Patient-14 | √ |  | √ |  |
| patient-15 |  |  | √ |  |
| patient-16 |  |  | √ |  |
| patient-17 | √ |  | √ |  |
| patient-18 | √ |  | √ |  |
| patient-19 |  | √ |  |  |
| patient-20 |  |  | √ |  |
| patient-21 | √ |  | √ |  |
| patient-22 | √ | √ |  |  |
| patient-23 | √ |  | √ |  |
| patient-24 | √ |  | √ |  |
| patient-25 | √ |  |  |  |
| patient-26 |  |  | √ |  |
| patient-27 |  |  | √ |  |
| patient-28 | √ |  | √ |  |
| patient-29 | √ |  |  |  |
| patient-30 |  | √ | √ |  |
| patient-31 |  |  | √ |  |
| patient-32 | √ |  |  |  |
| patient-33 | √ |  |  | √ |
| patient-34 | √ |  | √ |  |
| patient-35 |  |  | √ |  |
| patient-36 |  |  | √ |  |
| patient-37 |  |  | √ |  |
| Note:   1. ≥ 50% reduction of seizure duration; 2. less impaired consciousness during seizures; 3. prominent improvement in the post-ictal state; 4. disappearance of clustered seizures. | | | | |

| Table S2. Predictors for successful ASM withdrawal on multivariable logistic regression (n = 56) | | |
| --- | --- | --- |
| Variables retained in the model | OR (95% CI) | *P* value |
| Epilepsy type (generalized vs. focal) | 0.30 (0.08-1.15) | 0.079 |
| Number of ASMs at the start of KDT | 1.52 (0.62-3.73) | 0.360 |
| Response to KDT at 3 months (responders vs. non-responders) | 0.53 (0.14-1.92) | 0.332 |
| Abbreviation: ASM, anti-seizure medication; KDT, ketogenic diet therapy; OR, odds ratio; CI, confidence interval. | | |
